# Supplementary material for: Neoadjuvant PARP inhibitor scheduling in BRCA1 and BRCA2 related breast cancer: PARTNER, a randomized phase II/III trial
Source: Nat Commun. 2025 May 13;16:4269. doi: 10.1038/s41467-025-59151-0 (PMC12075821; doi:10.1038/s41467-025-59151-0)
Supplement: Supplementary file 2 — Reporting Summary [file 41467_2025_59151_MOESM2_ESM.pdf]

nature portfolio

Corresponding author(s): Jean E Abraham

Last updated by author(s): Mar 19, 2025

Reporting Summary

Nature Portfolio wishes to improve the reproducibility of the work that we publish. This form provides structure for consistency and transparency in reporting. For further information on Nature Portfolio policies, see our Editorial Policies and the Editorial Policy Checklist.

Statistics

For all statistical analyses, confirm that the following items are present in the figure legend, table legend, main text, or Methods section.

n/a	Confirmed	
☐	☒	The exact sample size (n) for each experimental group/condition, given as a discrete number and unit of measurement
☒	☐	A statement on whether measurements were taken from distinct samples or whether the same sample was measured repeatedly
☐	☒	The statistical test(s) used AND whether they are one- or two-sided<br>*Only common tests should be described solely by name; describe more complex techniques in the Methods section.*
☐	☒	A description of all covariates tested
☐	☒	A description of any assumptions or corrections, such as tests of normality and adjustment for multiple comparisons
☐	☒	A full description of the statistical parameters including central tendency (e.g. means) or other basic estimates (e.g. regression coefficient) AND variation (e.g. standard deviation) or associated estimates of uncertainty (e.g. confidence intervals)
☐	☒	For null hypothesis testing, the test statistic (e.g. F, t, r) with confidence intervals, effect sizes, degrees of freedom and P value noted<br>*Give P values as exact values whenever suitable.*
☒	☐	For Bayesian analysis, information on the choice of priors and Markov chain Monte Carlo settings
☒	☐	For hierarchical and complex designs, identification of the appropriate level for tests and full reporting of outcomes
☒	☐	Estimates of effect sizes (e.g. Cohen's d, Pearson's r), indicating how they were calculated

Our web collection on statistics for biologists contains articles on many of the points above.

Software and code

Policy information about availability of computer code

Data collection	Clinical data were collecting using Elsevier's MACRO database.
Data analysis	All statistical analyses were carried out in R (v4.1.0) .

For manuscripts utilizing custom algorithms or software that are central to the research but not yet described in published literature, software must be made available to editors and reviewers. We strongly encourage code deposition in a community repository (e.g. GitHub). See the Nature Portfolio guidelines for submitting code & software for further information.

Data

Policy information about availability of data

All manuscripts must include a data availability statement. This statement should provide the following information, where applicable:

- Accession codes, unique identifiers, or web links for publicly available datasets
- A description of any restrictions on data availability
- For clinical datasets or third party data, please ensure that the statement adheres to our policy

Source data files have been provided with this article. The data regarding the baseline patient information, survival outcomes, the trial protocol and other detailed therapeutic information have been provided as Supplementary information and within the Article. De-identified data collected in the PARTNER study will be made available to researchers whose full proposal for their use of the data has been approved by the PARTNER Trial Management Group, and whose research includes a clear and comprehensive research plan with statistical considerations adequately completed. The data required for the approved, specified purposes and the trial protocol will be provided after completion of a data sharing agreement. Data sharing agreements will be set up by the trial steering and management groups and

will include clear instructions on publication, reporting and usage policy. A minimum dataset of anonymised data will be made available after full publication of the trial and related work. Source data files used for the article have been provided. A copy of the PARTNER trial protocol is available in the supplementary information. Requests for data should be addressed to ja344@cam.ac.uk.

Research involving human participants, their data, or biological material

Policy information about studies with human participants or human data. See also policy information about sex, gender (identity/presentation), and sexual orientation and race, ethnicity and racism.

Reporting on sex and gender	The study focused on breast cancer patients. Although gender or sex identifying information was not collected, it is likely that the majority of participants in this study were assigned female at birth.
Reporting on race, ethnicity, or other socially relevant groupings	This study included all participants who met eligibility criteria for the study with no data collected on race, ethnicity, or other socially relevant grouping.
Population characteristics	Patients aged between 16 and 70 years with histologically confirmed stage T1-4, N0-3 (tumour or axillary lymph node diameter ≥ 10mm) invasive breast cancer, confirmed HER2 negative, and Eastern Cooperative Oncology Group performance status (ECOG PS) 0-1 were eligible. All patients had mandatory gBRCA testing at trial entry. Detailed eligibility criteria are provided in the methods section.
Recruitment	Participants were assessed for eligibility after consent at 23 UK centres between June 2016 to May 2023. All eligible participants were randomised to the study.
Ethics oversight	This research complies with all relevant ethical regulations. Preclinical in vivo studies complied with all relevant ethical regulations for animal testing and research, followed AstraZeneca's global bioethics policy, and received ethical approval from the AstraZeneca ethical committee. The HBCx-17, HBCx-10 and HBCx-9 PDX model studies were carried out at XenTech, France in accordance with French regulatory legislation concerning the protection of laboratory animals. The PARTNER trial protocol (NCT03150576 and EudraCT: 2015-002811-13) was approved by Northwest - Haydock Research Ethics Committee (ref: 15/NW/0926) and the trial was performed in accordance with the Declaration of Helsinki and the European Clinical Trials Directives 2001/20/EC.

Note that full information on the approval of the study protocol must also be provided in the manuscript.

Field-specific reporting

Please select the one below that is the best fit for your research. If you are not sure, read the appropriate sections before making your selection.

[x] Life sciences   [ ] Behavioural & social sciences   [ ] Ecological, evolutionary & environmental sciences

For a reference copy of the document with all sections, see nature.com/documents/nr-reporting-summary-flat.pdf

Life sciences study design

All studies must disclose on these points even when the disclosure is negative.

Sample size	In this gBRCAm cohort, a total of 178 patients were needed to achieve a 90% power with a 5% significance level, assuming the pCR rate of 55%in control group and 75% in research group. Considering a non-compliance of 5%, it was planned to recruit a total of 188 gBRCAm patients between the control and the selected research group. The study design included an interim analysis for futility reported in the results of this manuscript. The trial was stopped at the interim analysis point on the advice of the independent data monitoring and safety committee (IDMSC).
Data exclusions	The treatment effect was estimated using a population defined based upon the mITT principle, while the safety of the experimental treatment and all other analyses included patients who had at least one dose of trial treatment.
Replication	The main analysis was performed by another independent statistician and check against the results of the trial statistician. No discrepancies were found. Two pathologists independently reviewed the slides.
Randomization	The trial design and dosing schedules are discussed in the main text. Patients were randomised using minimisation method in a 1:1:1 ratio in Stage 1 and Stage 2 with a web-based central randomisation system.
Blinding	This is an open label study. The pathologists were blinded to the treatment arm.

Reporting for specific materials, systems and methods

We require information from authors about some types of materials, experimental systems and methods used in many studies. Here, indicate whether each material, system or method listed is relevant to your study. If you are not sure if a list item applies to your research, read the appropriate section before selecting a response.

Materials & experimental systems

n/a	Involved in the study
☒	☐ Antibodies
☒	☐ Eukaryotic cell lines
☒	☐ Palaeontology and archaeology
☐	☒ Animals and other organisms
☐	☒ Clinical data
☒	☐ Dual use research of concern
☒	☐ Plants

Methods

n/a	Involved in the study
☒	☐ ChIP-seq
☐	☒ Flow cytometry
☒	☐ MRI-based neuroimaging

Animals and other research organisms

Policy information about studies involving animals; ARRIVE guidelines recommended for reporting animal research, and Sex and Gender in Research

Laboratory animals	Male rats, strain RccHan:WIST, age 12 weeks. Female nude mice, strain HSD:Athymic Nude-Foxn1nu, age 6-9 weeks.
Wild animals	The study did not involve wild animals
Reporting on sex	Male rats were chosen as we knew the doses to get correct exposures in males. Female rats give a different level of exposure.
Field-collected samples	The study did not involve samples collected from the field
Ethics oversight	All in vivo studies complied with all relevant ethical regulations for animal testing and research, followed AstraZeneca's global bioethics policy and received ethical approval from the AstraZeneca ethical committee.<br>HBCx-17 PDX study was carried out at XenTech, France in accordance with French regulatory legislation concerning the protection of laboratory animals.

Note that full information on the approval of the study protocol must also be provided in the manuscript.

Clinical data

Policy information about clinical studies

All manuscripts should comply with the ICMJEguidelines for publication of clinical research and a completedCONSORT checklist must be included with all submissions.

Clinical trial registration	NCT03150576 and EudraCT: 2015-002811-13
Study protocol	Available in supplementary materials.
Data collection	Participants were assessed for eligibility after consent at 23 UK centres between June 2016 to May 2023.
Outcomes	The primary endpoint was pathological response (pCR), and secondary endpoints included event-free (EFS), overall survival (OS),) breast cancer specific survival (BCSS), relapse free survival (RFS), breast cancer specific survival (BCSS), distant disease-free survival (DDFS), local recurrence-free survival (LRFS), time to second cancer (TTSC), residual cancer burden (RCB), quality of life (QoL) and safety.

Plants

Seed stocks	*Report on the source of all seed stocks or other plant material used. If applicable, state the seed stock centre and catalogue number. If plant specimens were collected from the field, describe the collection location, date and sampling procedures.*
Novel plant genotypes	*Describe the methods by which all novel plant genotypes were produced. This includes those generated by transgenic approaches, gene editing, chemical/radiation-based mutagenesis and hybridization. For transgenic lines, describe the transformation method, the number of independent lines analyzed and the generation upon which experiments were performed. For gene-edited lines, describe the editor used, the endogenous sequence targeted for editing, the targeting guide RNA sequence (if applicable) and how the editor was applied.*
Authentication	*Describe any authentication procedures for each seed stock used or novel genotype generated. Describe any experiments used to assess the effect of a mutation and, where applicable, how potential secondary effects (e.g. second site T-DNA insertions, mosiacism, off-target gene editing) were examined.*

Flow Cytometry

Plots

Confirm that:

☒ The axis labels state the marker and fluorochrome used (e.g. CD4-FITC).

☒ The axis scales are clearly visible. Include numbers along axes only for bottom left plot of group (a 'group' is an analysis of identical markers).

☒ All plots are contour plots with outliers or pseudocolor plots.

☒ A numerical value for number of cells or percentage (with statistics) is provided.

Methodology

Sample preparation	Bone marrow analysis<br>Rat femurs were removed at necropsy and both ends were trimmed. Bone marrow cells were immediately flushed out with 3 ml PBS containing 50% foetal calf serum (FCS) using 3-ml syringe and a microlance 3 needle (BD). Cell suspension was syringed and filtered through 100 µm strainer, collected by centrifugation (300 g/ 7 min/ 4ºC) and washed once in HBSS containing 2% FCS and 10 mM HEPES (staining buffer). Total cell count of isolated cells was determined by automated cell counter (Countess, Invitrogen). Cell concentration was adjusted to 1x107 cells/ ml in staining buffer and processed for antibody staining. For the CD90.1 and Lineages cocktail, 1 ml of cell suspension was resuspended in 100 µl staining buffer containing anti-rat CD90.1-APC (dilution 1:100), CD6-FITC (1:100), CD3-FITC (1:100), CD11b-FITC (1:200), Granulocytes-FITC (1:200) from BD Pharmingen and CD45RC-FITC (1:100) purchased from Serotec. For the CD71 and CD45 cocktail, 100 µl cell suspension was resuspended in 100 µl staining buffer containing anti-rat CD71-FITC (dilution 1:10) and CD45-PE (1:10) from Serotec. Cells were incubated with antibodies for 30 minutes at room temperature (RT) and washed twice in staining buffer. After the final centrifugation, 1 ml of staining buffer was added to CD90.1-Lineages stained cells. For cell pellet staining with CD71-CD45 antibodies, resuspension was in 200 µl staining buffer. This was followed by the addition of 10 µl of LDS-751 cell-permeant nuclear stain (Life Technologies). Cells were incubated in the dark for 30 minutes prior to flow cytometry analysis.<br><br>In vitro cell line analysis<br>SUM149PT cells were seeded in 6 cm dishes (NUNC) (150,000 cells/dish) 72 hour prior to treatment and then treated with olaparib (1 mM) and carboplatin (10 mM) according to the schedules outlined in Figure 6 and Supplementary. Figure 11. On day four, cells were EdU treated for 30 minutes, detached using Tryple Express (Gibco), fixed with 2% paraformaldehyde (PBS, Themro Scientific), and then permeabilised and EdU labelled according to the immunofluorescence protocol described above. Cells were resuspended in block buffer containing 1 mg/ml DAPI, and live and single cells were gated. Ten thousand DAPI-positive cells (Supplementary Figure S11) were analysed with the LSRFortessa Cell Analyser (BD, 647799s0). Cell numbers of the different stages of the cell cycle - gap 1 (G1), DNA synthesis (S) and the gap 2 and mitosis (G2/M) phases, were quantified using FlowJo Engine v5 (BD) software to establish the relative proportion of cells within these phases before and following treatment.
Instrument	BD FACS Aria II (bone marrow), LSR Fortessa Cell Analyser (BD, 647799s0) (cell line work)
Software	FlowJo
Cell population abundance	Not applicable to our studies as we did not perform any sorting of the cells
Gating strategy	Total bone marrow cells were first analysed on forward and side scatter (FSC-A vs SSC-A), single cells were selected based on FSC-H vs FSC-W properties. Untreated unstained cells (negative control) and single positive controls for individual fluorophores (APC-conjugated CD90.1 vs FITC-conjugated lineage cocktail) were used to define APC-positive/ FITC-negative gate to quantify the population of interest.

☒ Tick this box to confirm that a figure exemplifying the gating strategy is provided in the Supplementary Information.
